# Interstitial Keratitis with Panuveitis: A Rare Manifestation of Ocular Tuberculosis

**DOI:** 10.4269/ajtmh.26-0264

**Published:** 2026-09-29

**Authors:** Sayali A. Karande, Somasheila I. Murthy

**Affiliations:** Shantilal Shanghvi Eye Institute, Mumbai, India

A 14-year-old Indian girl presented with a 1-week history of bilateral ocular redness and decreased vision. Best-corrected visual acuity was 20/320p in the right eye and 20/200 in the left eye. Slit-lamp examination showed stromal corneal infiltrates along the leading edge (right eye: 12–3 o’clock, 5 × 3 mm; left eye: 1 o’clock, 3 × 5 mm), with adjacent nebular scarring (right eye: 8–12 o’clock curvilinear fashion; left eye: 3 × 4-mm paracentral) and superficial vascularization. No overlying epithelial defect, loss of corneal sensations, or fluorescein staining was noted. There was bilateral 2+ anterior chamber inflammation, posterior synechiae, and posterior subcapsular cataract. Fundus evaluation showed grade 1 vitritis and bilateral grade 3 disc edema with cystoid macular edema (Figure 1). She reported a similar episode 1 year earlier limited to corneal involvement and granulomatous anterior uveitis, which resolved with topical corticosteroid therapy without significant visual loss. Systemic workup demonstrated a strongly positive Mantoux test (20 × 18 mm), positive QuantiFERON-TB Gold: (Qiagen, Germantown, USA) assay, and enlarged paratracheal lymph nodes on high-resolution computed tomography scan; treponemal serology and HIV ELISA were negative. Despite polymerase chain reaction being the gold standard, the diagnosis of tubercular granulomatous panuveitis was guided by the patient’s ethnicity, presentation of granulomatous uveitis with interstitial keratitis and supporting laboratory and radiological findings. She was treated with weight-based pediatric anti-tubercular therapy consisting of isoniazid (10 mg/kg per day), rifampicin (15 mg/kg per day), pyrazinamide (35 mg/kg per day), and ethambutol (20 mg/kg per day) for 2 months followed by isoniazid and rifampicin at the same doses for 4 months along with a tapering course of systemic corticosteroids (1 mg/kg body weight) and topical 1% prednisolone acetate. At 2 weeks, best corrected visual acuity improved to 20/30 partial in both eyes, with resolution of disc edema and cystoid macular edema (Figure 1). By 3 months, the IK resolved, leaving residual nebular scarring at 1 o’clock (Figure 1). No recurrence was noted at 1 year.

**Figure 1.**
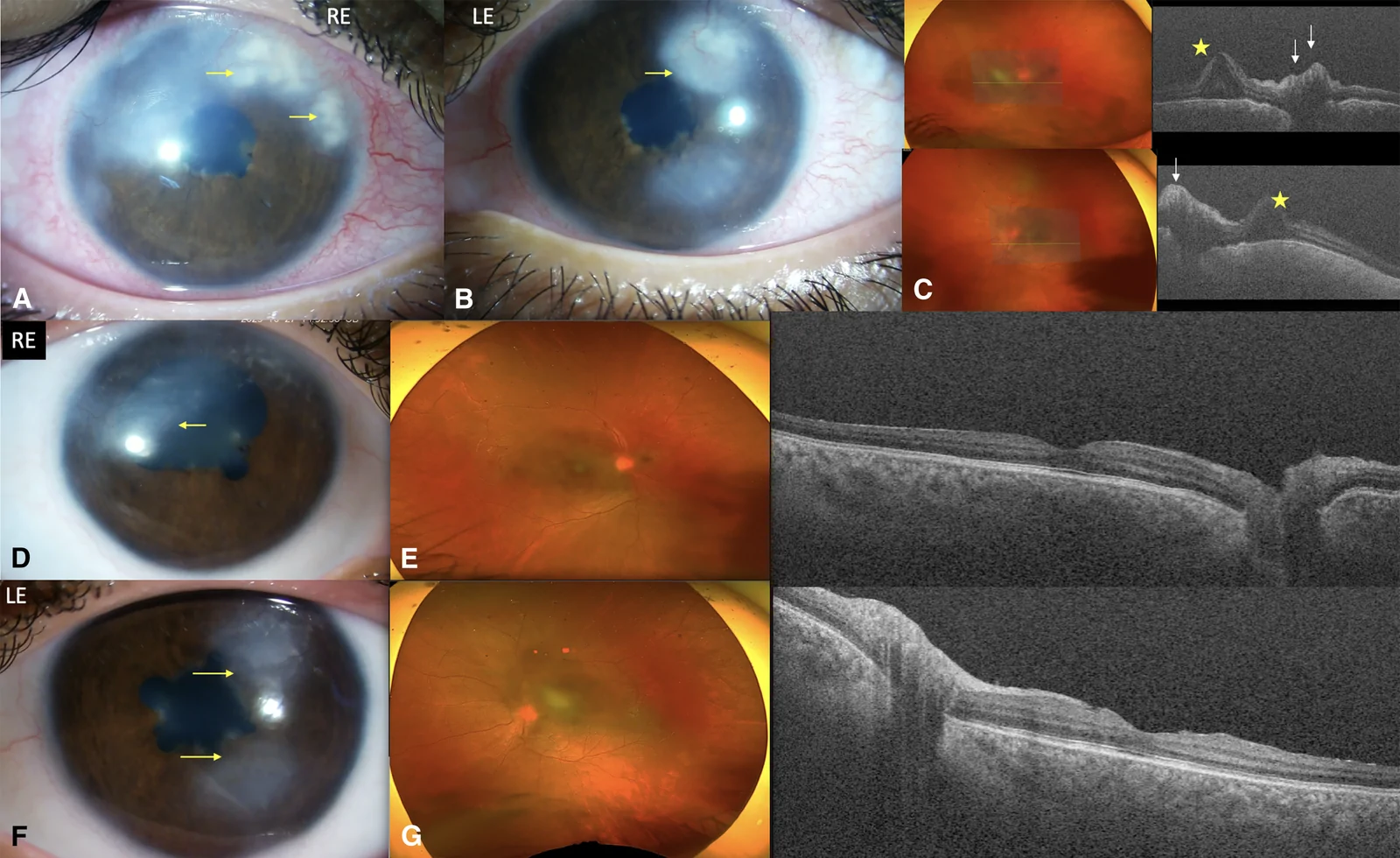
(**A**) At presentation, the slit section of the right eye shows conjunctival congestion, corneal infiltrates (12–3 o’clock; yellow arrows) with a residual scar, and complicated cataract. (**B**) Diffuse illumination of the left eye shows conjunctival congestion, corneal infiltrate (1 o’clock; yellow arrow) with vascularization, and a residual scar. (**C**) Wide-field fundus imaging and optical coherence tomography (OCT) of both eyes shows disc edema (white arrows) with cystoid macular edema (yellow stars). (**E** and **G**) At 2 weeks, wide-field fundus imaging and OCT show resolution of disc edema and cystoid macular edema. (**D** and **F**) At 3 months, diffuse slit lamp images of both eyes show resolution of interstitial keratitis with a residual macular scar (yellow arrows).

Corneal involvement in ocular tuberculosis remains an uncommon but clinically important manifestation, with reported presentations including phlyctenulosis, IK, and peripheral ulcerative keratitis with or without associated anterior uveitis.^1^ Our patient demonstrated bilateral tubercular panuveitis in conjunction with IK, a combination that has not been previously described, thereby expanding the known spectrum of ocular tuberculosis. Interstitial keratitis is postulated to arise from a delayed hypersensitivity response to mycobacterial antigens, resulting in sterile corneal infiltration rather than direct microbial invasion.^2^ This immunopathogenic mechanism underscores the need for a high index of suspicion, particularly in endemic regions, as isolated steroid therapy predisposes to recurrences and subsequent complications, such as secondary glaucoma and complicated cataract.^3^

Given the persistent global burden of tuberculosis and its protean ocular manifestations, early recognition and institution of appropriate antitubercular therapy alongside judicious immunosuppression are critical.^4^ Timely diagnosis not only aids in preventing recurrent inflammation but also, mitigates the risk of irreversible visual morbidity.
